# Neurocysticercosis in Radiographically Imaged Seizure Patients in U.S. Emergency Departments^1^

**DOI:** 10.3201/eid0806.010377

**Published:** 2002-06

**Authors:** Samuel Ong, David A. Talan, Gregory J. Moran, William Mower, Michael Newdow, Victor C.W. Tsang, Robert W. Pinner, the EMERGEncy ID NET Study Group

**Affiliations:** * Olive View-University of California at Los Angeles Medical Center, Sylmar, California, USA; † Centers for Disease Control and Prevention, Atlanta, Georgia, USA

**Keywords:** neurocysticercosis, seizure

## Abstract

Neurocysticercosis appears to be on the rise in the United States, based on immigration patterns and published cases series, including reports of domestic acquisition. We used a collaborative network of U.S. emergency departments to characterize the epidemiology of neurocysticercosis in seizure patients. Data were collected prospectively at 11 university-affiliated, geographically diverse, urban U.S. emergency departments from July 1996 to September 1998. Patients with a seizure who underwent neuroimaging were included. Of the 1,801 patients enrolled in the study, 38 (2.1%) had seizures attributable to neurocysticercosis. The disease was detected in 9 of the 11 sites and was associated with Hispanic ethnicity, immigrant status, and exposure to areas where neurocysticercosis is endemic. This disease appears to be widely distributed and highly prevalent in certain populations (e.g., Hispanic patients) and areas (e.g., Southwest).

Neurocysticercosis is the most common parasitic disease of the central nervous system. It is endemic in many developing countries and has been cited as the primary reason that “epilepsy” is twice as common in these countries as in more industrialized nations such as the United States (1). The prevalence of neurocysticercosis in some of these developing countries exceeds 10% (2,3), where it accounts for up to 50% of cases of late-onset epilepsy (4).

International travel and immigration are bringing neurocysticercosis to areas where it is not endemic. Several case series have been published from a variety of institutions throughout the United States, especially in the Southwest (5–10), but none has directly assessed the prevalence of neurocysticercosis. Domestic acquisition of the disease has been documented not only in large, metropolitan centers that attract large numbers of immigrants but also in less urban areas of North and South Carolina (11). Local acquisition has even been demonstrated in such unlikely areas as an Orthodox Jewish community, where it was attributed to the employment of domestic workers from Central and South American countries (12).

Seizures are the most frequent, and often the only, clinical manifestation of neurocysticercosis; they occur in 70% to 90% of cases (10,13). Because seizure patients frequently go to emergency departments, we chose this setting to perform a prospective study to determine the prevalence and epidemiology of this disease.

## Materials and Methods

This study was a prospective case series of patients who visited any of a network of 11 geographically diverse, university-affiliated, urban emergency departments (*EMERGE*ncy ID NET) from July 1996 to September 1998. The approximate total annual visit census of these emergency departments is 900,000. Institutional review board approval for the study was obtained at all sites. A more detailed description of *EMERGE*ncy ID NET, including its administration and the processes of data transfer and compilation, has been published (14).

Emergency department patients >5 years of age were enrolled in the study if they had a known or suspected seizure and had undergone neuroimaging, either computed tomography scanning (CT) or magnetic resonance imaging (MRI). Patients <5 years of age were excluded to avoid enrolling a potentially large number of patients with febrile seizures. The treating physician recorded demographic and clinical data including age, sex, race, immigrant status, foreign travel, prior seizure history, seizure type, CT and MRI findings, presumptive diagnosis, and disposition.

When blood was drawn from a patient as part of the evaluation, an additional tube was obtained for this study. Serum specimens from 890 of the 1,801 patients enrolled were sent to the Centers for Disease Control and Prevention so that serologic testing for cysticercosis could be performed. Serum samples were tested by enzyme-linked immunoelectrotransfer blot for *Taenia solium–*specific antibodies, as described (15,16). Briefly, this assay uses seven purified glycoprotein antigens from larval cysts of *T. solium*, namely, GP50, GP42-39, GP24, GP21, GP18, GP14, and GP13, where the prefix GP stands for glycoprotein and the number indicates the molecular mass in kilodaltons. These antigens are used in an immunoblot format to detect infection-specific antibodies. Reactions to at least one antigen band are considered positive (15,16).

On the basis of a classification scheme proposed by Del Brutto (17), our case definition for neurocysticercosis required either 1) CT scan finding(s) characteristic of neurocysticercosis (i.e., multiple calcifications or multiple cystic lesions) with or without a positive serologic test, or 2) CT scan finding(s) consistent with neurocysticercosis (i.e., a single cystic, calcified, or hypodense lesion) and a positive serologic test. Radiologists at each site read CT scans without regard for or knowledge of the study. Study coordinators at each site then abstracted the absence or presence of findings relevant to the study from the radiology reports. Simple descriptive statistics were used to summarize the clinical features of patients with and without neurocysticercosis. Relative risk ratios (RRs) and their corresponding 95% confidence intervals (CIs) were determined by Fisher’s exact test.

## Results

A total of 1,801 eligible patients with 1,833 emergency department visits were enrolled during the 2-year study period. Twenty-eight patients had multiple visits; four patients underwent evaluation on three separate occasions (Table 1). A diverse group of seizure patients were enrolled in the study (Table 2).

**Table 1:** Demographic and clinical characteristics of 1,833 neuroimaged emergency department seizure patients^a^

Characteristics	Patients (%)
Sex (male)	1,220 (67)
Race/ethnicity	
Black	753 (41)
White, non-Hispanic	643 (35)
Hispanic	320 (17)
Native American	43 (2.3)
Asian/Pacific Islander	33 (1.8)
Other or unknown	41 (2.2)
Insurance	
Medicare/private	462 (25.2)
Medicaid	391 (21.3)
Uninsured	762 (41.6)
Immigrant status^b^	
Born in USA	820 (61)
Not born in USA	178 (13)
Unknown	350 (26)
Exposure to disease-endemic region	
No travel outside USA	950 (51.8)
Exposure to disease-endemic region	342 (18.7)
Unknown travel history	541 (29.5)
Seizure type	
Generalized tonic/clonic	1,577 (86)
Focal motor	114 (6.2)
Partial complex	86 (4.7)
Unknown or undocumented	56 (3.1)
Seizure history	
Prior seizure history	810 (44)
No prior seizure history	896 (49)
Unknown seizure history	127 (7)

^a^The median age (interquartile range) in yrs for these patients was 40 (range 30–51 yrs). ^b^1,348 patients; immigrant status data was not collected on the first 485 patients.

**Table 2:** Emergency department physicians’ diagnoses for 1,348^a^ neuroimaged seizure patients

Diagnosis	Seizure patients (%)
Etiology uncertain	515 (38)
Alcohol or drug abuse/withdrawal	253 (19)
Head injury	105 (7.8)
Epilepsy	92 (6.8)
Other^b^	104 (7.7)
Brain tumor	42 (3.1)
Metabolic disorder (e.g., hypoglycemia)	39 (2.9)
Stroke	36 (2.7)
Neurocysticercosis	30 (2.2)
Nontraumatic cerebral hemorrhage	22 (1.6)
Syncope, possibly not seizure	25 (1.9)
Meningitis or brain abscess	18 (1.3)
Pseudoseizure	14 (1.0)
Toxoplasmosis	12 (0.9)
No diagnosis documented	42 (3.1)

^a^The method of categorizing discharge diagnoses was modified during the study. These data represent the last 1,348 of 1,833 patient encounters. ^b^Other category includes six patients with recent neurosurgery, three with toxic levels of anticonvulsant medications, three with reactions to non-anticonvulsant medication, three with systemic lupus erythematosus, and several other less frequently occurring causes.

From the entire study population, 2.1% (38) patients met the case definition for neurocysticercosis (Table 3). Of patients who underwent both CT scanning and serologic testing, 2.9% met the case definition. Thirty-four patients satisfied the case definition based on classic CT scan findings, and four satisfied the case definition based on a positive serologic test coupled with CT scan findings consistent with neurocysticercosis. Neurocysticercosis was identified at 9 of the 11 study sites; 6 sites enrolled more than one patient (Table 3). Patients with this disease tended to be younger than patients who did not meet the case definition (Table 4). Patients were also more likely to be Hispanic, have been born outside the United States, have visited or lived in an endemic region, be uninsured, and have a reported history of neurocysticercosis. Overall, approximately 9% of patients with Hispanic ethnicity who came to an emergency room with a seizure met the case definition for neurocysticercosis. The prevalence of the disease in the Hispanic patients with seizures ranged from 9% to 13.5% in the highest risk sites.

**Table 3:** Neurocysticercosis and selected demographic characteristics of seizure patients, U.S. sites

Site	Total seizure patients enrolled	Hispanic patients enrolled (%)	Immigrants enrolled^a^ (%)	Neurocysticercosis patients identified (%)
Albuquerque, NM	107	58 (54)	9 (8)	6 (5.6)
Atlanta, GA	146	4 (3)	6 (4)	0 (0.0)
Charlotte, NC	300	11 (4)	17 (6)	4 (1.3)
Kansas City, MO	164	12 (7)	3 (2)	1 (0.6)
Los Angeles, CA	91	52 (57)	21 (23)	9 (9.9)
New Orleans, LA	174	9 (5)	8 (5)	2 (1.1)
New York, NY	184	50 (27)	54 (29)	1 (0.5)
Orlando, FL	68	6 (9)	9 (13)	0 (0.0)
Philadelphia, PA	185	20 (11)	19 (10)	1 (0.5)
Phoenix, AZ	243	90 (37)	27 (11)	10 (4.1)
Portland, OR	171	8 (5)	5 (3)	4 (2.3)
	1,833	320 (17)	179 (10)	38 (2.1)
^a^Immigration data were not obtained from the first 490 patients enrolled.				

**Table 4:** Demographic and clinical characteristics of neurocysticercosis patients^a^ and non-neurocysticercosis patients

Features	Neurocysticercosis patients n=37 (%)	Non-neurocysticercosis patients n=1,796 (%)		Relative risk 95% CI
Sex, male	27 (73.0)	1,189 (66.0)		
Racial/ethnic background^b^				
Black	4 (10.8)	746 (41.6)		
White, non-Hispanic	3 (8.1)	640 (35.7)		
Hispanic	29 (78.4)	291 (16.2)	17.1 (7.9 to 37.1)	
Insurance status				
Medicare/private	7 (18.9)	455 (25.3)		
Medicaid	3 (8.1)	386 (21.5)		
Uninsured	22 (59.5)	738 (41.1)	2.5 (1.2 to 5.2)	
Immigrant status^c^				
Born in US	5 (21.0)	815 (62.0)		
Not born in US	12 (50.0)	166 (13.0)	11.1 (3.9 to 31.0)	
Unknown	7 (29.0)	343 (26.0)		
Exposure to endemic region				
No travel out of US	0 (0)	950 (52.9)		
Exposure to endemic region	28 (75.7)	314 (17.5)	158 (9.7 to 2,581)	
Unknown travel history	9 (24.3)	532 (29.6)		
Prior history of neurocysticercosis^c^				
Positive prior history	3 (16.0)	5 (0.5)	21.6 (7.8 to 59.8)	
No prior history	16 (84.0)	906 (99.5)		
Seizure type				
Generalized	26 (70.3)	1,551 (86.4)	0.38 (0.18 to 0.80)^d^	
Tonic/clonic	4			
Focal motor	2 (5.4)	112 (6.2)		
Partial complex	7 (18.9)	79 (4.4)		
Unknown/undocumented	2 (5.4)	54 (3.0)		
Seizure history				
New onset	19 (51.0)	877 (49.0)	1.1 (0.56 to 2.02)	
Prior seizure history	17 (46.0)	793 (44.0)		
Serologic testing				
Seropositive	9 (36.0)	9 (1.0)	NA^e^	
Seronegative	16 (64.0)	856 (99.0)		
Disposition				
Admission	16 (43.0)	865 (48.0)	1.0 (0.5 to 2.0)	
Discharge	15 (41.0)	801 (45.0)		

^a^A patient was a person who met the case definition for neurocysticercosis. See text. The median age for neurocysticercosis patients was 32 yrs, with a range of 25–44 yrs; the median age of non-neurocysticercosis patients was 40 yrs (range 30–52 yrs). ^b^n = 36 for this category. ^c^n = 1,343; immigrant status was not collected from the first 490 patients enrolled. ^d^Generalized seizure versus focal motor or partial complex seizures. ^e^NA, not applicable. Comparison was not done since serology was part of the case definition for neurocysticercosis. CI, confidence intervals.

Patients meeting the case definition for neurocysticercosis were not more likely to have a new-onset seizure (versus having an established history of seizures; RR 1.1; CI 0.56 to 2.01). Neurocysticercosis patients were, however, more likely to have focal motor or partial complex seizures than those without neurocysticercosis (RR 2.6; CI 1.3 to 5.5) (Table 4).

Serologic testing was performed on 49.4% (890) of the 1,801 patients enrolled, and results were positive in 2% (18) cases. No significant differences were detected in age, seizure history, seizure type, prior history of neurocysticercosis, immigrant status, or exposure to an endemic region between the patients who underwent serologic testing and those who did not. Nine of the seropositive patients had CT scan findings consistent with neurocysticercosis; nine did not. Compared with the seronegative patients, seropositive patients were more likely to be Hispanic (RR 8.7; CI 3.1 to 24.1), have visited or lived in a neurocysticercosis-endemic region (RR 6.8; CI 2.2 to 20.5), and have an abnormal CT scan (RR 1.7; CI 1.2 to 2.3). Seropositivity was not significantly associated with either new-onset seizures or prior seizure history. Serology was positive in 27% of the patients who had neuroimaging findings characteristic of neurocysticercosis, 5.3% of those with consistent neuroimaging findings, and 1.1% of those with neuroimaging findings inconsistent with neurocysticercosis.

Seven patients reported a history of neurocysticercosis. Four (57%) of these patients had neuroimaging findings typical of or consistent with neurocysticercosis. Serologic testing was performed on two of the seven patients with one negative and one positive result.

The use of our case definition identified two patients who were not diagnosed with neurocysticercosis by the emergency department physician but who were later clinically diagnosed with neurocysticercosis. One was a child born in the United States to Laotian parents whose travel history was unknown; the other was a man of Hispanic ethnicity whose travel history and immigration status were not available. On the other hand, half of the patients diagnosed with neurocysticercosis by their treating physician did not satisfy our case definition. No confirmed cases of domestically acquired neurocysticercosis were identified during the study period.

## Discussion

Immigrants continue to make up an increasing proportion of the U.S. population. By some reports, they will constitute up to 60% of the new immigrants and new births over the next few decades (18,19). By the year 2037, Latinos will outnumber whites as the dominant ethnic group in California, mirroring a population surge that is sweeping across the Southwest (20). Because neurocysticercosis is endemic in many of the countries from which these persons are emigrating, its presence in the United States reflects these immigration trends.

Previous etiologic surveys of seizures in the United States and other industrialized countries have focused on brain tumors, strokes, and birth defects (21–23). Infections such as toxoplasmosis and meningitis or meningoencephalitis constitute a small minority of causes in seizure patients. More recently, HIV and its attendant complications have become prominent causes of adult-onset seizures (23). Neurocysticercosis, while a prominent cause of seizures in less developed nations, has not appeared in these studies.

An increasing number of neurocysticercosis cases (5–10) have been reported throughout the United States, which suggests that the prevalence of this disease may be on the rise. Because these previous case series were conducted retrospectively, primarily through chart reviews over periods as long as a decade, understanding the epidemiology and impact of neurocysticercosis is difficult. To our knowledge, this prospective study is the first to address the prevalence of neurocysticercosis in seizure patients in the United States.

Neurocysticercosis was identified at 9 of 11 sites and was responsible for 2.1% of seizures overall. In some sites, e.g., Los Angeles, California, and Albuquerque, New Mexico, the prevalence was nearly 10%. That neurocysticercosis has not appeared in previous U.S. studies on the epidemiology of seizures and now appears in our study as the cause of up to 10% of seizures in some areas suggests a substantial increase in frequency of this disease. Another study from a Los Angeles–area hospital corroborates this finding: 12% of the seizures seen in the authors’ emergency department were attributable to neurocysticercosis (24).

Previous reports on neurocysticercosis in the United States, mostly retrospective case series, have focused on the clinical and epidemiologic aspects of the disease (5–10) Those studies are somewhat limited by the inadequacies and incompleteness inherent in retrospective data collection. It also seems problematic when epidemiologic information (e.g., exposure to a disease-endemic area) constitutes part of the case definition/inclusion criteria (e.g., exposure to cysticercosis-endemic area) but then epidemiologic information is subsequently reported as a result (e.g., percentage of patients who had visited an area where cysticercosis is endemic). In contrast, our data were collected at the time of evaluation, and our case definition was based solely on clinical criteria. As such, our study provides additional corroboration to the findings of previous studies reporting strong associations between neurocysticercosis and Hispanic ethnicity, immigrant status, and prior exposure to disease-endemic regions. Neurocysticercosis patients were also more likely to be uninsured; however, lack of insurance was also associated with being Hispanic and an immigrant. Consistent with previous studies, most neurocysticercosis patients did come to the emergency department with a generalized tonic clonic seizure, but such patients were more likely to have focal motor or partial complex seizures than were the seizure patients without neurocysticercosis. On the basis of our results, neurocysticercosis must be strongly considered in emergency department seizure patients of Hispanic descent since nearly 1 in 10 were affected, a figure that was even higher in certain areas.

From 1988 through 1990, 7.2% of neurocysticercosis cases reported to the Los Angeles Department of Health Services were locally acquired (25). The rate of domestic acquisition has been even higher (17% to 26%) in some studies of pediatric neurocysticercosis (6,8). These rates of domestic acquisition appear to have increased from earlier studies in the late 1970s and early 1980s, when the rates were in the range of 2% to 3%. Because years can pass before symptoms develop, the incidence of domestically acquired cases will likely continue to rise.

The apparent increase in the prevalence of neurocysticercosis carries a substantial economic impact. Nearly half of the seizure patients in our study were admitted to the hospital. The average cost of hospitalization for seizures in one study was $1,615 per patient, not including physician charges (26). The economic toll extends beyond such direct costs. Compared with the general population, seizure patients are seen in the emergency departments 2½ times more frequently, admitted to the hospital 3 times more frequently, and treated by specialists 3 times more frequently; they also receive psychological counseling 7 times more frequently (27) These figures still underestimate the true economic impact of neurocysticercosis because up to 30% of patients who visit an emergency room do not have seizures but rather a variety of other neurologic symptoms such as headache, visual changes, ataxia, and confusion. Hydrocephalus may develop in a substantial number if patients, requiring neurosurgical intervention.

Any study of neurocysticercosis is limited by the difficulty in clearly establishing the diagnosis. The only true measure for the diagnosis of neurocysticercosis is brain biopsy, which is clearly impractical. This study therefore implemented a case definition that incorporates the classification scheme proposed by Del Brutto (17) but is therefore limited by the predictive value of CT scan and serology. Serology has previously been demonstrated to be sensitive in cases with multiple cysts (94%) but less sensitive with single cysts or calcified lesions (28%) (28). Specimen storage and periodic bulk mailing may have further affected intrinsic test performance.

We found a considerable discrepancy between patients who were diagnosed with neurocysticercosis by their physicians and patients who met our case definition. Because we did not specifically ask the treating physicians how they arrived at their diagnosis, the exact reasons for this discrepancy are unclear. However, emergency room physicians appear to rely considerably on epidemiologic information when diagnosing neurocysticercosis. Of patients diagnosed by their physicians, 98% were Hispanic (compared with our 76%) and 80% were immigrants (compared with our 50%). Ten percent of the patients with physician-diagnosed neurocysticercosis had normal CT scans.

Additional limitations to the study include the fact that the participating network sites are university-affiliated emergency departments. This fact may limit the generalizability of our results to other patient populations. Ideally, serologic testing would have been performed on all patients, but laboratory testing is not routinely performed for all seizure patients seen in emergency departments. Patients who did undergo serologic testing were, however, not statistically different from those who did not, on the basis of the demographic information collected.

In summary, while neurocysticercosis accounts for a small proportion (2.1%) of all seizures in university-affiliated, U.S. emergency department patients, its geographic distribution appears diverse; the highest concentration is in the Southwest and in Hispanics. Our observations are consistent with current immigration trends that suggest the growing importance of neurocysticercosis in the United States. Continued surveillance and further studies of screening and treatment strategies appear warranted.

## References

[1] International League Against Epilepsy. Relationship between epilepsy and tropical disease. Epilepsia. 1994;35:89–93. 10.1111/j.1528-1157.1994.tb02916.x 8112262

[2] Bern C, Garcia HH, Evans C, Gonzalez AE, Verastegui M, Tsang VC, Magnitude of the disease burden from neurocysticercosis in a developing country. Clin Infect Dis. 1999;29:1203–9. 10.1086/313470 10524964 PMC2913118

[3] Sarti E, Schantz PM, Plancarte A, Wilson M, Gutierrez IO, Lopez AS, Prevalence and risk factors for *Taenia solium* taeniasis and cysticercosis in humans and pigs in a village in Morelos, Mexico. Am J Trop Med Hyg. 1992;46:677–85. 1621892 10.4269/ajtmh.1992.46.677

[4] Medina MT, Rosas E, Rubio-Donnadieu F, Sotelo J. Neurocysticercosis as the main cause of late-onset epilepsy in Mexico. Arch Intern Med. 1990;150:325–7. 10.1001/archinte.150.2.325 2302008

[5] Richards FO, Schantz PM, Ruiz-Tiben E, Sorvillo FJ. Cysticercosis in Los Angeles County. JAMA. 1985;254:3444–8. 10.1001/jama.254.24.3444 4068185

[6] Mitchell WG, Crawford TO. Intraparenchymal cerebral cysticercosis in children: diagnosis and treatment. Pediatrics. 1988;82:76–82. 3288959

[7] McCormick GF. Cysticercosis—review of 230 patients. Bull Clin Neurosci. 1985;50:76–101. 3842090

[8] Rosenfeld EA, Byrd SE, Shulman ST. Neurocysticercosis among children in Chicago. Clin Infect Dis. 1996;23:262–8. 8842261 10.1093/clinids/23.2.262

[9] Scharf D. Neurocysticercosis. two hundred thirty-eight cases from a California hospital. Arch Neurol. 1988;45:777–80. 3291833 10.1001/archneur.1988.00520310087022

[10] Shandera WX, White AC Jr, Chen JC, Diaz P, Armstrong R. Neurocysticercosis in Houston, Texas. a report of 112 cases. Medicine. 1994;73:37–52. 10.1097/00005792-199401000-00004 8309361

[11] Centers for Disease Control. Locally acquired neurocysticercosis—North Carolina, Massachusetts, and South Carolina, 1989–1991. MMWR Morb Mortal Wkly Rep. 1992;41:1–4. 1727973

[12] Moore AC, Lutwick LI, Schantz PM, Pilcher JB, Wilson M, Hightower AW, Seroprevalence of cysticercosis in an Orthodox Jewish community. Am J Trop Med Hyg. 1995;53:439–42. 7485700 10.4269/ajtmh.1995.53.439

[13] White AC Jr. Neurocysticercosis: a major cause of neurological disease worldwide. Clin Infect Dis. 1997;24:101–15. 10.1086/513661 9114131

[14] Talan DA, Moran GJ, Mower WR, Newdow M, Ong S, Slutsker L, *EMERGE*ncy ID NET: An emergency department-based emerging infections sentinel network. Ann Emerg Med. 1998;32:703–11. 10.1016/S0196-0644(98)70071-X 9832668

[15] Tsang VCW, Brand JA, Boyer AE. An enzyme-linked immunoelectrotransfer blot assay and glycoprotein antigens for diagnosing human cysticercosis (*Taenia solium*). J Infect Dis. 1989;159:50–9. 2909643 10.1093/infdis/159.1.50

[16] Garcia HH, Gilman RH, Catacora M, Verastegui M, Gonzales AE, Tsang VCW, Serological evolution of neurocysticercosis patients after antiparasitic therapy. J Infect Dis. 1997;175:486–8. 9203680 10.1093/infdis/175.2.486 PMC3025428

[17] Del Brutto OH, Wadia NH, Dumas M, Cruz M, Tsang VC, Schantz PM. Proposal of diagnostic criteria for human cysticercosis and neurocysticercosis. J Neurol Sci. 1996;142:1–6. 10.1016/0022-510X(96)00130-X 8902711

[18] Howard RUS. population boom to create real estate need. Los Angeles Times 1999 Nov 2.

[19] Facts & figures (immigrants from Latin America; middle-class Hispanics) [brief report]. Hispanic. 2001;14:16.

[20] Alvarez F. Dawn of a new majority. Los Angeles Times 1999 Oct 17.

[21] Hauser WA, Annegers JF, Kurland LT. Incidence of epilepsy and unprovoked seizures in Rochester, Minnesota: 1935–1984. Epilepsia. 1993;34:453–68. 10.1111/j.1528-1157.1993.tb02586.x 8504780

[22] Eisner RF, Turnbull TL, Howes DS, Gold IW. Efficacy of a “standard” seizure workup in the emergency department. Ann Emerg Med. 1986;15:69–75. 10.1016/S0196-0644(86)80483-8 3942354

[23] Sempere AP, Villaverde FJ, Martinez-Menendez B, Cabeza C, Pena P, Tejerina JA. First seizure in adults: a prospective study from the emergency department. Acta Neurol Scand. 1992;86:134–8. 10.1111/j.1600-0404.1992.tb05054.x 1414222

[24] Henneman PL, DeRoos F, Lewis RJ. Determining the need for admission in patients with new onset seizures. Ann Emerg Med. 1994;24:1108–14. 10.1016/S0196-0644(94)70240-3 7978592

[25] Sorvillo FJ, Waterman SH, Richards FO, Schantz PM. Cysticercosis surveillance: locally acquired and travel-related infections and detection of intestinal tapeworm carriers in Los Angeles County. Am J Trop Med Hyg. 1992;47:365–71. 1524150 10.4269/ajtmh.1992.47.365

[26] Dias MS, Carnevale F, Li V. Immediate posttraumatic seizures: is routine hospitalization necessary? Pediatr Neurosurg. 1999;30:232–8. 10.1159/000028803 10461069

[27] Wiebe S, Bellhouse DR, Fallahay C, Eliasziw M. Burden of epilepsy: the Ontario health survey. Can J Neurol Sci. 1999;26:263–70. 10563210 10.1017/s0317167100000354

[28] Wilson M, Bryan RT, Fried JA, Ware DA, Schantz PM, Pilcher JB, Clinical evaluation of the cysticercosis enzyme-linked immunoelectrotransfer blot in patients with neurocysticercosis. J Infect Dis. 1991;164:1007–9. 1940452 10.1093/infdis/164.5.1007

